# Mechanism of Pipeline Leakage Sound Generation and Leak Detection Technology Under Multiple Operational Conditions

**DOI:** 10.3390/s25237281

**Published:** 2025-11-29

**Authors:** Fei Chen, Taikeng Jiang, Latao Jiang, Chen Rong, Xiaohang Li, Liang Chen, Xuefei Xu, Jin Yang

**Affiliations:** 1 Fuqing Nuclear Power Co., Ltd., Sanshan Town, Fuqing 350318, China; chenfei@cnnp.com (F.C.);; 2 College of Optoelectronic Engineering, Chongqing University, Chongqing 400044, China; 3 College of Physics and Electronic Engineering, Chongqing Normal University, Chongqing 401331, China; 4 Xiamen Silicon Microelectronics Technology Co., Ltd., Unit 701, No. 370 Chengyi Avenue, Jimei District, Xiamen 361021, China

**Keywords:** acoustic emission, pipeline leakage detection, large eddy simulation, computational aeroacoustics, wavelet packet analysis, feature extraction, support vector machine

## Abstract

Acoustic emission detection technology is widely employed for leakage detection in water supply systems. However, this approach heavily relies on extensive field data to develop feature extraction and analysis models. Since field data cannot comprehensively cover all operational conditions—such as variations in pressure, pipe diameter, and leakage size—the limited generalization capability of these models often results in high rates of false negatives and false positives. To address these issues, this study utilizes Large Eddy Simulation (LES) to analyze leakage flow fields, establishing correlations between diverse operating conditions and flow field characteristics, including the areas of negative pressure zones and pressure pulsations. Based on these flow field findings, Computational Aeroacoustics (CAA) is applied to analyze the acoustic radiation field at leakage locations, thus clarifying the sound generation mechanisms of leakage-related acoustic signals, demonstrating strong agreement between simulation results and experimental data. Furthermore, wavelet packet energy ratio, centroid frequency, and frequency entropy are extracted as key feature parameters. A leakage detection model based on Support Vector Machine (SVM) is subsequently developed, achieving an accuracy of 98.6% across a wide range of operating conditions. This research enhances the capability for high-accuracy leakage detection with limited field data, offering valuable technical insights for the development of low-computation and low-hardware-cost leakage detection systems.

## 1. Introduction

Water supply systems are essential for delivering potable water to households and serve as the most cost-effective, efficient, and safe means of water distribution [1,2,3,4]. However, amid rapid urbanization in China, the total length of water supply pipelines has exceeded 1.3 million km, while the annual water supply has surpassed 60 billion cubic meters. More than 30% of these pipelines are aging, and leakage rates exceed 15%, leading to an annual water loss of approximately 9 billion cubic meters. Early detection of pipeline leaks is therefore crucial. Acoustic emission technology, a non-destructive and real-time monitoring method, facilitates rapid leak detection by analyzing acoustic signals generated by leakage. Owing to its high sensitivity and broad coverage, this technology has been extensively adopted for leakage detection in water supply networks [5,6,7,8,9].

Despite their potential, current leakage detection and acoustic emission technologies encounter substantial challenges in practical applications. The complex structure and diverse operational conditions of water supply systems lead to significant variability in acoustic signals induced by leakage. These variations are influenced by factors such as operating pressure, pipe material, fluid properties, and leakage aperture size, which complicate the extraction and recognition of reliable features [10,11,12,13]. Moreover, existing acoustic emission technologies rely heavily on extensive datasets of leakage events to develop robust models. However, the sporadic occurrence of leakage incidents and the complexity of data acquisition conditions make it difficult to collect sufficient field data covering a wide range of leakage scenarios. This limitation severely restricts the generalization capability of the models [14,15,16]. Additionally, the frequency bands of leakage acoustic signals vary significantly with operational conditions, and current sampling techniques often fail to strike an optimal balance between noise suppression and signal fidelity. This shortfall impedes efficient and accurate detection in complex environments [17].

Existing research has made notable progress in the analysis of pipeline leakage. For instance, Lu et al. investigated the flow field characteristics of urban pipeline leaks using a two-dimensional model, examining changes in gas pressure, velocity, and temperature under both steady and transient states. They observed that flow velocity increases with pressure and aperture size, while transient effects are negligible [18]. Martins et al. performed computational fluid dynamics (CFD) analysis on high-pressure pipeline leaks using three-dimensional grids, focusing on flow characteristics near leakage orifices and validating their findings through experimental studies [19]. Shan et al. explored diffusion patterns following natural gas pipeline ruptures [20], while Zhang et al. calculated flow field distributions for different orifice shapes using the Ffowcs Williams–Hawkings (FW-H) model [21]. Doshmanziari et al. proposed a model-driven approach for leak detection and localization in pipelines of finite length, utilizing multiphase flow simulation software to conduct numerical simulations of high-pressure pipeline leaks. The reliability of the method was subsequently validated through experimental studies. [22]. Liu et al. combined numerical simulations with field experiments to investigate the distributions of flow and acoustic fields, as well as the mechanisms of sound generation [23]. Papastefanou et al. analyzed vibration-acoustic signals from leaks in plastic pipes, highlighting the impact of aperture size and flow velocity on signal characteristics [24]. Mori et al. simulated aerodynamic noise in T-shaped rectangular pipes under varying flow rates, revealing the influence of fluid velocity on noise sources [25]. Recently, Li et al. proposed a hybrid modeling approach combining computational fluid dynamics (CFD) and genetic algorithm (GA) to optimize sensor placement for corrosion detection in pipelines, demonstrating the potential of integrating CFD with artificial intelligence in pipeline health monitoring [26]. While these studies provide valuable insights into fundamental flow and acoustic field patterns, they often lack analysis of sensor-monitored signals and fail to address adaptability to multi-condition scenarios, leaving practical applications underexplored.

To address these challenges, this paper proposes a CAA simulation-guided leak detection method, whose core innovation lies in directly integrating the physical insights obtained from high-fidelity flow–acoustic simulations into feature design and classification, thereby reducing reliance on large-scale field data.

Specifically, unlike existing studies where simulation and detection are often decoupled, this work combines Large Eddy Simulation (LES) in Fluent with vibro-acoustic analysis in LMS Virtual.Lab to systematically investigate leakage under a wide range of operating conditions—including pressure, pipe diameter, leak orifice size, and pipe material. The simulations reveal that, despite significant variations in these parameters, acoustic energy consistently concentrates in the 500–3000 Hz frequency band, and spectral characteristics—such as centroid frequency and signal regularity—exhibit systematic trends with leakage severity. This cross-condition stability of the dominant frequency band provides a reliable basis for feature selection. Accordingly, the selected features—wavelet packet energy ratio, centroid frequency, and frequency entropy—are not chosen empirically, but are strictly aligned with the dominant acoustic behaviors predicted by CAA. When combined with only 240 experimental samples to train an SVM classifier, the method achieves 98.6% detection accuracy, significantly outperforming purely data-driven approaches. These results validate the practical feasibility of a “simulation-informed, experiment-calibrated” strategy for real-world applications. This study offers a new pathway toward low-data-dependency intelligent monitoring of complex water supply networks, balancing physical interpretability with engineering practicality.

## 2. Analysis of Leakage Sound Generation Mechanism Based on Large Eddy Simulation and Aeroacoustics

### 2.1. Field Condition Analysis

To investigate the impact of boundary conditions on the characteristics of the flow field associated with leakage and the acoustic signals recorded on pipe walls, three-dimensional (3D) models were developed to represent various operational scenarios, with simulation parameters established in accordance with the respective boundary conditions. The detailed parameters are presented in Table 1 below.

In the flow field simulations, the primary focus is on the characteristics of the fluid. It is assumed that the pipeline is not affected by the flow field, and any deformation is negligible—indicating that pipeline material does not affect flow field characteristics. Additionally, the leakage aperture is considered sufficiently small relative to the pipeline, implying that the flow dynamics within the leakage hole have minimal effect on the overall flow field within the pipeline [23]. To comprehensively analyze the flow characteristics and the governing parameters of the flow field, the simulation encompasses the entire spectrum of operational conditions, with a particular emphasis on the flow field in the vicinity of the leakage hole. In the sound field simulation, typical operational conditions are selected to assess the acoustic signal characteristics of the pipeline wall, with corresponding conditions chosen for experimental validation.

### 2.2. Theoretical Basis of Flow and Acoustic Field Simulation

When the leakage occurs in the water supply pipeline, the fluid near the leakage sprays out at high speed driven by the pressure gradient, which produces noise and vibration, stimulates the vortex-induced vibration of the pipe wall, and at the same time, the cavitation collapse will also produce additional noise. The main sound sources of pipeline leakage include the coupling effects of jet-induced pipeline vibration, turbulent vibration and cavitation noise. This study employs the Large Eddy Simulation (LES) technique to analyze the transient flow field [27], which is more resource-efficient compared to other methods and can effectively capture the details of turbulence. Compared with RANS, LES can capture transient vortical structures and pressure fluctuations that dominate acoustic generation, while remaining computationally feasible compared with DNS. The characteristic turbulence scale of the leakage jet is on the order of the 1 mm orifice diameter, corresponding to dominant vortex shedding frequencies below 5 kHz. This makes LES particularly suitable for generating transient pressure and velocity fields for acoustic field coupling. The Smagorinsky–Lilly model was adopted as the subgrid-scale (SGS) model to represent the effects of unresolved small-scale eddies. According to Lighthill’s acoustic analogy theory [28], sound sources can be classified into monopoles, dipoles, and quadrupoles, with dipole sources primarily arising from the vibrations of the pipe walls. Considering that sensors typically detect the vibrations of the pipe walls, this paper focuses on analyzing the acoustic-structural coupling between the leakage flow field and the pipe wall.

### 2.3. Simulation Settings for Flow Field and Sound Field

In this paper, 20 groups of steady-state flow fields with different pipe pressures, pipe inner diameters and leakage defect types were simulated based on Fluent CFD.

The operational conditions used in this study were defined according to typical pipeline working scenarios, including different internal pressures, pipe diameters, and leakage defect types. The fluid inside the pipeline was water at ambient temperature, and the pipe material could be adjusted according to the required working condition. The external pressure at the leakage hole was assumed to be 0.1 MPa (atmospheric pressure).

**Figure 1:**
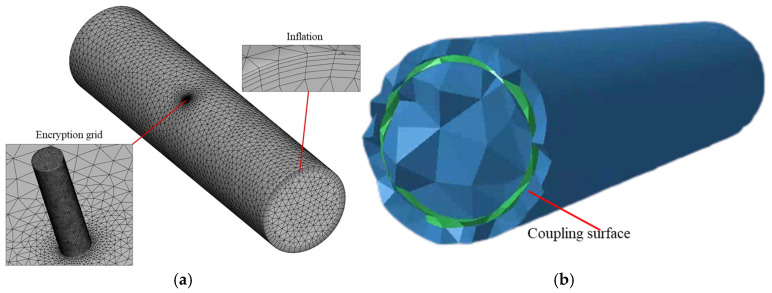
Flow field and sound field grid division. (**a**) Flow field grid; (**b**) Sound field grid.

Since the pipeline itself does not affect the flow field inside the pipeline, the calculational domain is the internal flow channel of the pipeline In order to capture the flow characteristics within the pipeline, as shown in Figure 1a, a tetrahedral mesh is used with local refinement at the leakage points. The pipeline inlet is configured as a velocity inlet with a flow rate of 4 m/s, while the outlet is set as a pressure outlet with pressure equal to the pipeline’s internal operating pressure. The leakage hole is set to 0.1 MPa atmospheric pressure. The realizable k-ε turbulence model and energy equation were used for steady-state flow simulation. The steady-state results are used in the subsequent large eddy simulation (LES) to calculate the transient flow field. The transient simulation used a time step of 1 × 10^−4^ s with 2000 steps, which satisfies the Courant–Friedrichs–Lewy (CFL) requirement (CFL < 0.5) and ensures sufficient temporal resolution for resolving pressure fluctuations up to 5000 Hz. To ensure spatial accuracy, local mesh refinement and boundary-layer grids were applied around the leakage orifice. A mesh-independence study was also performed using the DN100 pipeline with a 1 mm leakage orifice. As shown in Table 2, The results showed that once the mesh exceeded approximately three million elements, the variations in key flow parameters were below 3%, confirming mesh convergence; this mesh density was therefore adopted for all LES simulations.

Twelve sets of working conditions were selected for the sound field calculation. The computational domain included the fluid domain, the pipe model and the radiated external sound field. The fluid was water, the pipe materials were B235 carbon steel, stainless steel and PU, and the external sound field was air. As shown in Figure 1b, the mesh of the radiated sound field met the requirement that the maximum element edge length was less than 1/6 of the shortest wavelength. The coupling between the flow field and the acoustic field was implemented through a one-way fluid–structure–acoustic transfer. Specifically, the transient pressure fluctuations on the pipe wall obtained from the LES results in Fluent were exported as boundary excitations and imported into LMS Virtual.Lab for acoustic field computation. The data were mapped to the structural surface mesh using consistent nodal coordinates to ensure accurate correspondence between the CFD and acoustic domains. The outer pipe surface was defined as a vibro-acoustic radiation boundary, and the surrounding air region was modeled as a far-field acoustic domain with an impedance boundary condition. This indirect coupling approach was selected because it effectively preserves the unsteady pressure characteristics of the flow while avoiding the high computational cost associated with fully coupled CFD–CAA simulations. The frequency range to be solved was 1–5000 Hz, with a frequency resolution of 10 Hz. The calculation results were the frequency spectrum curves of the monitoring points, and the vibration characteristics of the pipe wall were analyzed in detail.

## 3. Results and Discussion of Leakage Flow Field and Sound Field Simulation

### 3.1. Flow Field Simulation Analysis

In the flow field calculation results, this study selects pressure and velocity contour maps to reflect the actual conditions of the flow field. However, the macroscopic contour maps cannot fully reflect the regularities brought about by different operating conditions to a certain extent. Therefore, we chose to compare the pressure and velocity changes along the vertical centerline of the leakage hole to analyze the field variations under different conditions. Additionally, we selected the negative pressure area and mass flow rate to reflect the overall sound pressure level trends under different operating conditions. The trends in the contour maps are consistent, and this paper only presents the contour maps under different pressures; contour maps for other operating conditions can be found in the contour maps for the other operating conditions are not shown here for brevity but follow the same trends.

**Figure 2:**
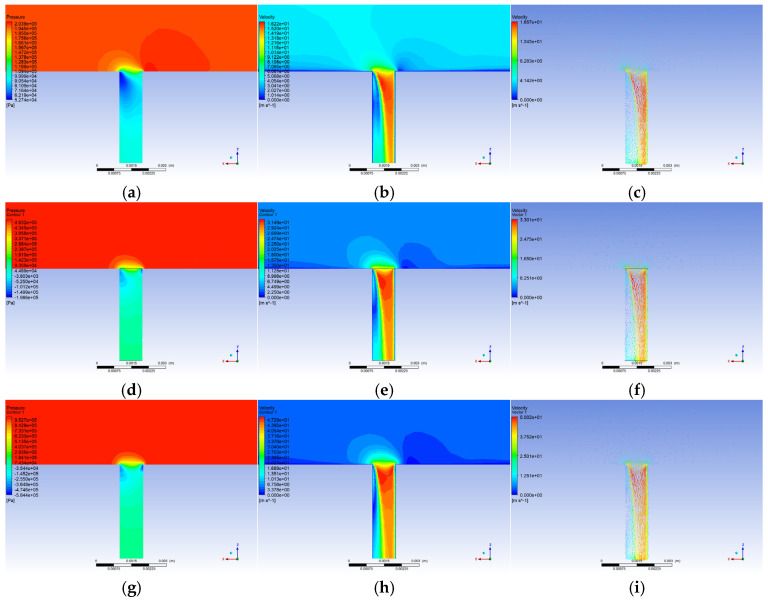
Flow field pressure, velocity contour and Velocity vector. (**a**) 0.2 MPa Pressure contour; (**b**) 0.2 MPa Velocity contour; (**c**) 0.2 MPa Velocity vector; (**d**) 0.5 MPa Pressure contour; (**e**) 0.5 MPa Velocity contour; (**f**) 0.5 MPa Velocity vector; (**g**) 1 MPa Pressure contour; (**h**) 1 MPa Velocity contour; (**i**) 1 MPa Velocity vector.

As shown in Figure 2, when the pipeline leaks, the leak hole communicates with the atmosphere, and the fluid in the pipe forms a rapid pressure drop near the leak hole, resulting in a significant pressure gradient. The pressure at the end of the leak hole drops to atmospheric pressure, and the negative pressure zone on both sides is asymmetrical: the left side has a large range but a small degree, and the right side has a small range but a high degree. Behind the leakage hole, there is a local weak pressure drop due to the flow back. Although the overall fluid velocity within the pipeline remains uniform and aligns with the inlet velocity, it experiences a rapid increase as it exits the leak, resembling a jet-like flow, which is subsequently moderated by the influence of the adjacent negative pressure zones. The trajectory of the fluid ejection is angled, with backflow occurring in the negative pressure region on the left side, and the low-pressure area behind the leak contributing to a localized decrease in fluid velocity.

**Figure 3:**
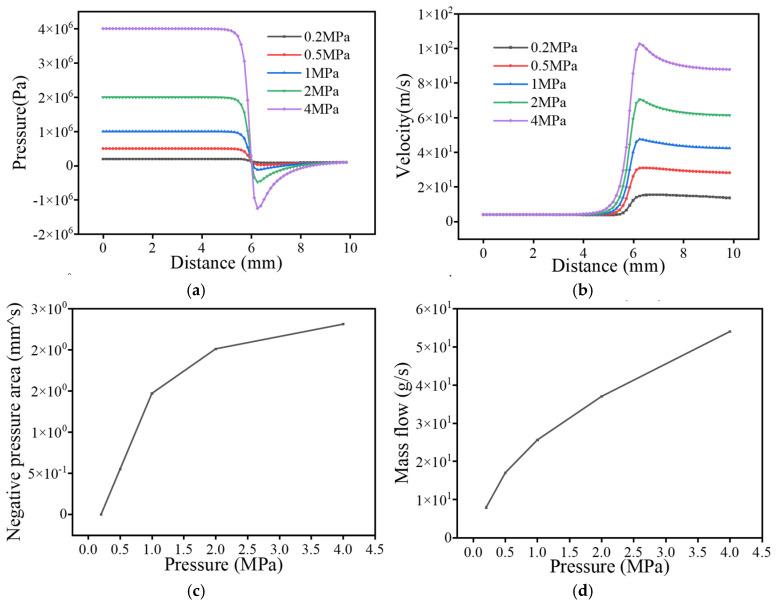
Diagram of the flow field under different pressure. (**a**) Pressure change curve; (**b**) Velocity curve; (**c**) Negative pressure area change curve; (**d**) Mass flow change curve.

As shown in Figure 3, the leakage–pipe junction is identified as the initiation region of pressure decay, and the attenuation magnitude increases significantly with the rise in internal pressure. The minimum pressure decreases from 0.4 kPa at 0.2 MPa to −5.8 kPa at 4 MPa, while the negative-pressure region expands from 0 mm^2^ to 2.47 mm^2^. Meanwhile, the velocity evolution exhibits a consistent trend with the pressure distribution: the peak jet velocity increases from approximately 16.9 m/s at 0.2 MPa to 107 m/s at 4 MPa, showing an enhancement factor of more than six times. This pressure–velocity coupling phenomenon indicates that higher operating pressure leads to stronger jet momentum, enhanced shear-layer instability, and intensified turbulence–vortex interaction near the leakage orifice, which further enlarges the negative-pressure zone. Considering that the enlargement and persistence of negative pressure favor cavitation inception and interface-induced oscillations, this flow behavior implies a higher potential for low-frequency cavitation noise and periodic pulsation sources, whose acoustic manifestations will be quantitatively validated in the subsequent acoustic field simulation section.

**Figure 4:**
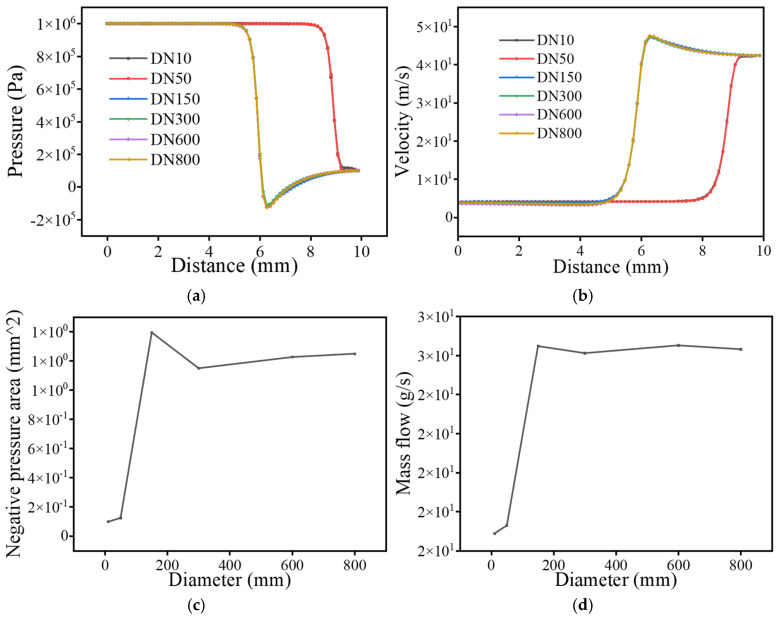
Diagram of flow field under different leakage hole size. (**a**) Pressure change curve; (**b**) Velocity curve; (**c**) Negative pressure area change curve; (**d**) Mass flow change curve.

As illustrated in Figure 4, the velocity evolution trend under different diameter-wall thickness combinations is primarily governed by wall thickness rather than the pipe diameter. When the wall thickness is 1 mm (DN10 and DN50), the leakage jet velocity rapidly rises to approximately 40 m/s and stabilizes thereafter. In contrast, for pipes with 3 mm wall thickness (DN150 and above), the velocity first peaks at around 45 m/s before stabilizing near 40 m/s. Correspondingly, the negative-pressure area increases from less than 0.2 mm^2^ to approximately 1 mm^2^, while mass flow rate rises from less than 20 g/s to nearly 30 g/s. These results indicate that jet momentum, shear stress, and turbulence intensity scale more strongly with local structural stiffness than with geometric diameter, implying a potential increase in high-frequency broadband radiation, to be verified in later acoustic analysis.

**Figure 5:**
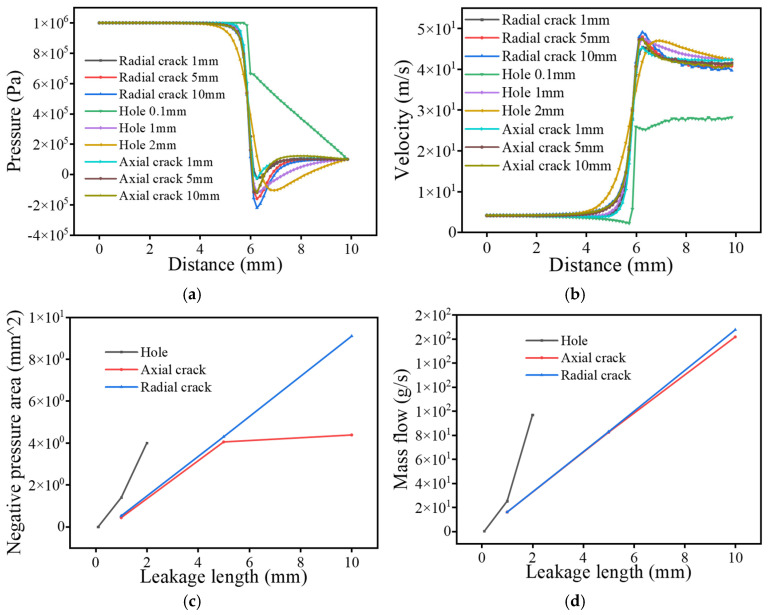
Diagram of flow field under different leakage hole type. (**a**) Pressure change curve; (**b**) Velocity curve; (**c**) Negative pressure area change curve; (**d**) Mass flow change curve.

As shown in Figure 5, all leakage orifice conditions exhibit a consistent rapid pressure-drop trend at the leak-pipe interface. However, small orifices (0.1 mm) demonstrate a slower and smoother pressure descent due to limited discharge area, while larger orifices experience a sharp drop to negative pressure over a very short distance before returning to atmospheric pressure. Correspondingly, the peak jet velocity increases from approximately 25 m/s to 42 m/s with larger apertures, accompanied by intensified turbulent fluctuations and stronger shear-driven instability. These results indicate that increasing orifice diameter enlarges the leakage jet scale and turbulence intensity, thereby enhancing both broadband and high-frequency acoustic radiation potential, with spectral differences to be quantified in the subsequent acoustic-field analysis.

In summary, the leakage characteristics of the pipeline are primarily determined by the pressure gradient, changes in flow velocity, and defect parameters. The rapid pressure drop and asymmetric negative pressure zone near the leak hole create significant flow field characteristics. The intense fluctuations in pressure and velocity can trigger cavitation effects and turbulence noise, with sound signals exhibiting enhanced low frequencies and high-frequency turbulence characteristics. The magnitude of the pressure affects the range of the negative pressure zone and the intensity of pressure fluctuations, while wall thickness and defect diameter directly determine flow velocity and turbulence characteristics, with thicker walls and larger diameters more easily exciting high-frequency signals. Overall, the parameters of the leak hole and the conditions of the pipeline dominate the spectral distribution and energy characteristics of the leakage sound signals, clarifying the intrinsic relationship between pressure, velocity, and sound signals, and providing an important theoretical basis for leak signal detection.

### 3.2. Sound Field Simulation Analysis

**Figure 6:**
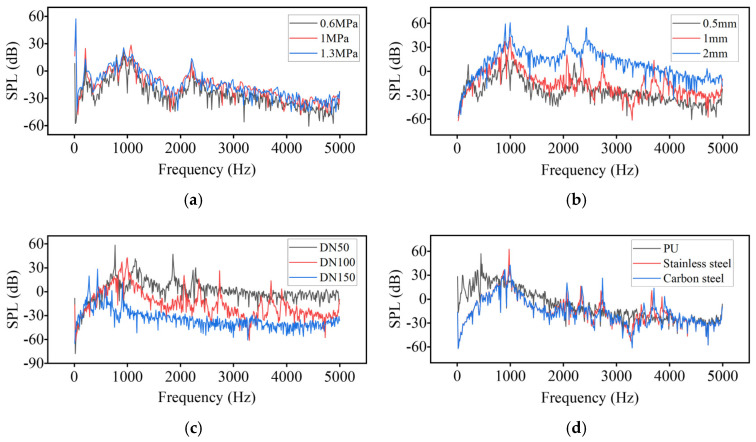
Simulated acoustic signals under different working conditions. (**a**) Spectrum of different pressures; (**b**) Spectrum of different leak hole sizes; (**c**) Spectrum of different pipe diameters; (**d**) Spectrum of different pipe materials.

As shown in Figure 6, the leakage-induced acoustic emission spectra demonstrate a broadband distribution predominantly within 500–3000 Hz, with multiple high-energy peaks concentrated between 500–2000 Hz, which is highly consistent with the underlying flow-field dynamics characterized by jet-induced shear-layer instability, turbulence energy cascade, and cavitation-related pressure pulsation mechanisms. Increasing internal pressure results in a notable amplification of spectral peak amplitudes without a significant shift in dominant frequency bands, suggesting that pressure primarily modulates the acoustic source strength rather than the source frequency structure. Enlarging the leakage orifice diameter enhances jet momentum flux and shear-induced turbulent vorticity, leading to elevated low-frequency spectral amplitudes and an extension toward higher frequency components. Variations in pipeline diameter reveal a scale-dependent spectral response, wherein larger diameters exhibit strengthened low-frequency components (<500 Hz) likely associated with larger structural modal participation and turbulence integral length scales, whereas smaller diameters favor higher-frequency components (1000–4000 Hz) consistent with reduced characteristic flow length scales and increased velocity gradients. Material dependency is also evident: polymeric (PU) pipes present low-frequency dominant responses with limited high-frequency radiation due to higher structural damping, whereas metallic pipes (B235 carbon steel and stainless steel) exhibit pronounced peaks near approximately 1000 Hz alongside enriched high-frequency components, attributable to stronger fluid–structure acoustic coupling and lower mechanical damping. Collectively, these observations confirm that the leakage spectral characteristics are physically governed by the scale-dependent dynamics of the leakage flow field, thereby providing a validated theoretical foundation for subsequent feature selection and machine-learning-driven diagnostic modeling.

By combining acoustic spectrum data and flow field characteristics under different pressures, pipe diameters, and leak aperture sizes, we can delve into the formation mechanisms of leak sound sources. An increase in pressure intensifies local flow field variations, resulting in enhanced turbulence and cavitation—thereby increasing the spectral energy of leakage noise. Although pipe diameter does not affect the flow field of micro-leaks, different diameters can impact the propagation of sound signals across different frequency bands. Larger diameters are more conducive to the propagation of lower-frequency noise, while smaller diameters lead to an increase in high-frequency noise. An increase in leak aperture directly affects the fluid’s jet flow rate and turbulence intensity, significantly raising the noise intensity, especially in the high-frequency range. Changes in mass flow rate essentially reflect variations in turbulence velocity within the flow field; an increase in flow rate results in a higher overall sound pressure level and tends to elevate the high-frequency range. Additionally, different pipe materials also affect the propagation of sound signals; PU materials facilitate the transmission of low-frequency signals, while metal pipes exhibit similar properties.

## 4. Multiple Operational Conditions Leakage Experiment and Acoustic Signal Characteristic Analysis

### 4.1. Construction and Data Collection of a Multiple Operational Conditions Leakage Experiment Platform

**Figure 7:**
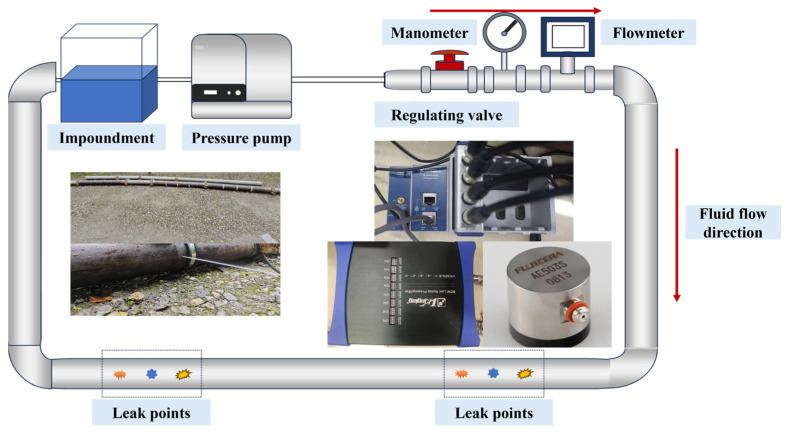
Multiple Operational Conditions Leakage Experiment Platform.

Based on the analysis of the flow field and sound field simulation, the mechanism by which the flow field generates sound signals has been clarified, and it has been revealed how the flow field affects the performance of sound signals through various parameters. To verify the correctness of the simulation sound signals for feature extraction guidance and to collect a dataset, a multi-condition simulated water supply pipe was constructed. A pipeline loop was built using equipment such as a reservoir and pump, as shown in Figure 7. The loop is constructed from B258 carbon steel, with an internal diameter of 100 mm. It can cover a pressure range of 0.5–1.5 MPa and maintain a flow rate of 4 m/s, which aligns with the parameters of a water supply pipeline branch. There are leakage holes ranging from 0.5 mm to 2 mm in the loop, and when the loop is filled with water, the leakage sound signals can be collected through sensors. The collection equipment uses a Fuji AE503S resonant sensor, which is amplified six times and transmitted to the host computer via an NI acquisition card. Previous sound field simulations indicated that leakage noise would occur below 5000 Hz, so a sampling rate of 20 KHz was defined, sampling once every second, with the sensor placed 5 cm from the leakage hole. To avoid introducing additional variability caused by leakage signal propagation attenuation and to ensure consistent signal quality across all operating conditions, the same sensor position was applied for all measurements. After data collection, the stored data will be subjected to signal analysis.

**Figure 8:**
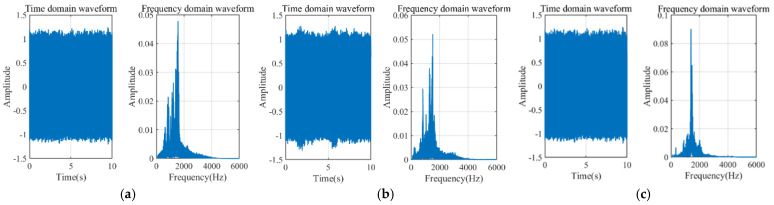
DN100, carbon steel, time-domain and frequency-domain graphs at different pressures with a thickness of 0.5 mm. (**a**) 0.6 MPa; (**b**) 1 MPa; (**c**) 1.3 MPa.

**Figure 9:**
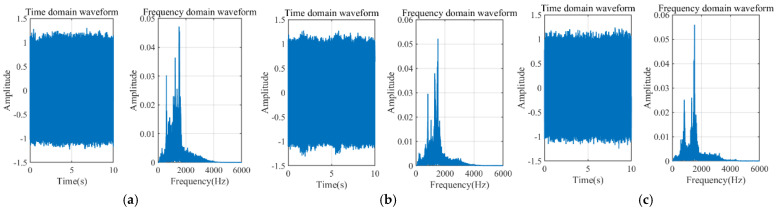
DN100, carbon steel, time-domain and frequency-domain graphs at 1 MPa with different aperture sizes. (**a**) 0.5 mm; (**b**) 1 mm; (**c**) 2 mm.

Figure 8 shows the leakage simulation circuit collected at different pressures of 0.6 MPa, 1 MPa, and 1.3 MPa, while Figure 9 displays the leakage signals at different aperture sizes of 0.5 mm, 1 mm, and 2 mm. It can be observed from the figures that the main frequency of the leakage signals is concentrated around 1000 Hz. However, there is low-frequency vibration noise caused by pump and pipeline vibrations in the low-frequency range, which is close to the leakage energy. Additionally, compared to the simulation, there is white noise introduced by the field environment and the data acquisition equipment.

### 4.2. Characteristic Parameter Analysis

**Figure 10:**
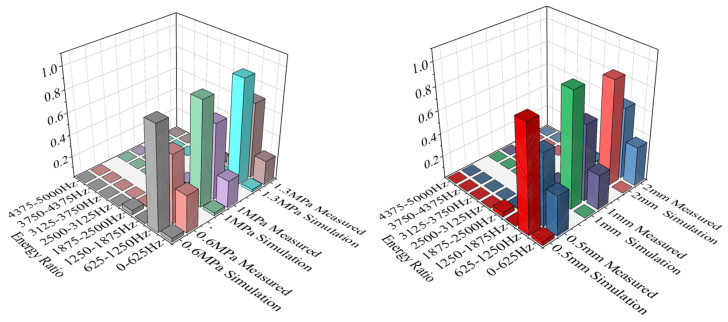
Energy proportion of wavelet packets with different pressures and different pore sizes.

**Figure 11:**
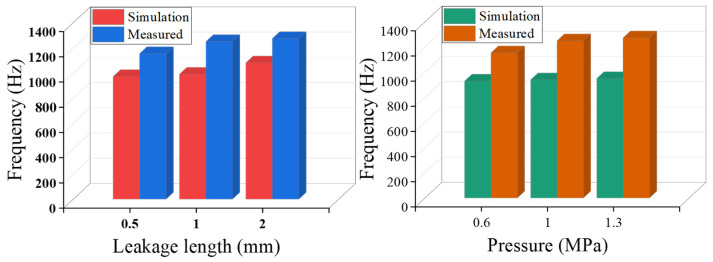
Center frequency at different pressures and different aperture sizes.

The frequency spectra of the acoustic field simulation signals presented in Figure 6 were systematically compared and analyzed against the corresponding experimentally measured signals shown in Figure 8 and Figure 9. Furthermore, the variations in the centroid frequencies of the wavelet packet energy under different pressure levels and leakage aperture sizes are summarized in Figure 10 and Figure 11. As shown in Figure 10, The analysis of the energy ratio of the signals extracted through wavelet packet transformation further confirmed the consistency in frequency domain characteristics between the simulated and measured signals. The Discrete Meyer (dmey) wavelet was used due to its good frequency localization in the low-frequency band; a three-level packet decomposition (eight ≈ 625 Hz subbands over 0–5 kHz) was applied, and optimal nodes were selected according to the maximum-energy criterion. From the perspective of frequency domain characteristics, the main frequency bands of both the simulated and measured signals are essentially the same, concentrated in the range of 500–3000 Hz. Although there is a deviation of about 200 Hz in the main frequency, this deviation is primarily caused by measurement errors of the experimental equipment, simplifications in boundary conditions, and the influence of ambient noise. The energy ratio of the low-frequency part of the signal is relatively high, which is related to low-frequency noise such as resonance and pump machinery present in actual working conditions, but the energy ratio of the peak part aligns well with the simulated signal. Additionally, as shown in Figure 11, the centroid frequency falls within the same frequency band. Despite the influence of background noise, which induces a slight shift toward higher frequencies, the overall energy distribution demonstrates a high degree of consistency between the simulated and measured results. distribution of the spectrum. More importantly, the simulation and experimental results exhibit highly consistent trends across different operating conditions, which strongly supports the physical fidelity of the CAA framework. Specifically: (i) As the internal pipe pressure increases, the spectral peak amplitude from the LES–acoustic simulation rises from 20.45 dB to 28.47 dB—an overall increase of approximately 39%—reflecting a continuous enhancement of acoustic source strength due to intensified turbulent jet dynamics. The measured signal under identical pressure variations shows a closely matching trend, with a peak amplitude increase of 35%. Although an absolute offset of about 200 Hz exists in centroid frequency between simulation and experiment—likely attributable to unmodeled structural damping or ambient noise—the relative change in centroid frequency with pressure exhibits a negligible error of only 0.4%. (ii) With increasing leak orifice size, the energy proportion in high-frequency sub-bands (e.g., 2500–5000 Hz) rises significantly: the simulation predicts a 195.7% increase, while the experimental data shows a comparable 187.7% growth, both indicating stronger turbulence and shear-layer instabilities at larger orifices. Again, despite a ~200 Hz absolute deviation in centroid frequency, the relative trend in frequency shift agrees within 0.6%. Collectively, these results demonstrate that—even though absolute spectral amplitudes and frequencies may differ slightly due to experimental noise, boundary simplifications, or material property uncertainties—the simulation accurately captures the essential acoustic behaviors: the dominant frequency band (500–3000 Hz), spectral energy distribution, centroid frequency evolution, frequency entropy, and, most critically, their systematic responses to parametric variations. This quantitative and qualitative consistency validates the use of CAA-predicted trends as reliable physical priors for feature engineering in leakage detection. Overall, the simulation results and the measured signals are highly consistent in terms of the main frequency band, spectral trends, centroid frequency, and frequency entropy, indicating that the established simulation model can effectively reflect the frequency domain characteristics of leakage signals under actual working conditions.

## 5. Leakage Detection Method and Field Application

### 5.1. Leakage Identification Method

**Figure 12:**
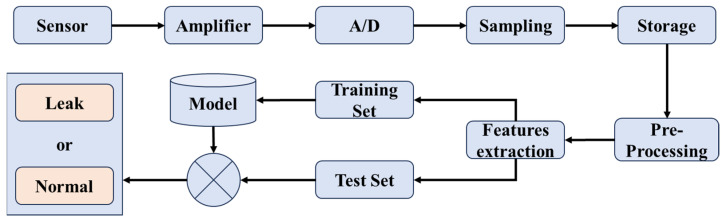
Data collection and training process.

Through the above analysis, it can be seen that the simulation method in this paper can effectively reflect the actual measured signals. It can extract features that fully represent the changes in leakage signals under various working conditions before actual testing, allowing for better integration of algorithms to identify leakage situations. This paper uses the SVM algorithm to build the leak detection model [29]. The specific steps for constructing the machine learning model are shown in Figure 12 below.

The data collected from the experiments undergoes preprocessing, including data cleaning, feature selection, and normalization. The main feature selected is the wavelet packet energy ratio, along with frequency domain features such as the centroid frequency and frequency entropy. These features were chosen not only for their empirical effectiveness but also for their strong physical correspondence with the leakage sound generation mechanisms revealed in Section 3 and Section 4: (i) The wavelet packet energy ratio captures the concentration of acoustic energy in the 500–3000 Hz band—a range that consistently dominates across various leakage conditions, including different internal pressures, orifice sizes, and pipe materials. This choice is further justified by the nature of leak-induced acoustic signals, which are non-stationary and span a broad frequency spectrum (0–5 kHz). The wavelet packet transform (WPT) provides superior resolution in the low-frequency region and enables precise energy decomposition across sub-bands, making it particularly well-suited for quantifying spectral energy distribution. Unlike the continuous wavelet transform (CWT), WPT does not require manual selection of mother wavelet scales and offers adaptive partitioning over the full frequency range; compared to empirical mode decomposition (EMD), it is more numerically stable and free from mode mixing. Guided by LES–CAA simulations and experimental results indicating that dominant acoustic energy resides within the 1–3 kHz interval, a three-level WPT decomposition was adopted, yielding sub-bands of 625 Hz width that effectively resolve this key energy region (see Figure 6); (ii) centroid frequency reflects spectral shifts driven by variations in jet velocity and turbulence intensity; and (iii) frequency entropy quantifies spectral regularity, effectively distinguishing structured leakage signals from random background noise.

A total of 240 datasets were divided into training and testing sets, with 80% of the data used for model training and 20% for evaluating model performance. Although the total sample size is modest, this is a low-dimensional classification task, and SVM is known to exhibit strong generalization capability under small-sample conditions—particularly when features are physically meaningful and well-structured. Moreover, the 240 samples are not redundant measurements; each originates from distinct operating conditions validated by both LES simulations and physical experiments, ensuring high signal-to-noise ratio and strong representativeness across the parameter space (including variations in pressure, orifice size, pipe material, and leakage status). Importantly, the dataset is balanced between leakage and non-leakage classes, avoiding bias due to class imbalance. To improve classification accuracy, this paper employed an SVM with an RBF kernel function and optimized hyperparameters (e.g., C and gamma) via grid search. During model development, 5-fold cross-validation was performed on the training set to assess stability and prevent overfitting. The cross-validation yielded an average accuracy of 98.1% with a standard deviation below 1.2%, indicating highly consistent performance across folds. The final model reported in this study corresponds to the fold with the highest validation accuracy, achieving 98.6% on the held-out test set—closely aligning with the cross-validated estimate and confirming that the model is robust and not overfitted.

To rigorously justify the selection of these features, an ablation study was conducted on the SVM classifier. As summarized in Table 3, the full feature set achieves a detection accuracy of 98.6%. Removing any single feature results in a noticeable performance drop—particularly when the wavelet packet energy ratio is excluded—confirming that the three features are complementary and collectively essential for robust leakage identification under diverse operational conditions. At the same time, the parameters extracted from the literature [30] were selected for comparison, and the proposed method shows superior generalization capability.

**Figure 13:**
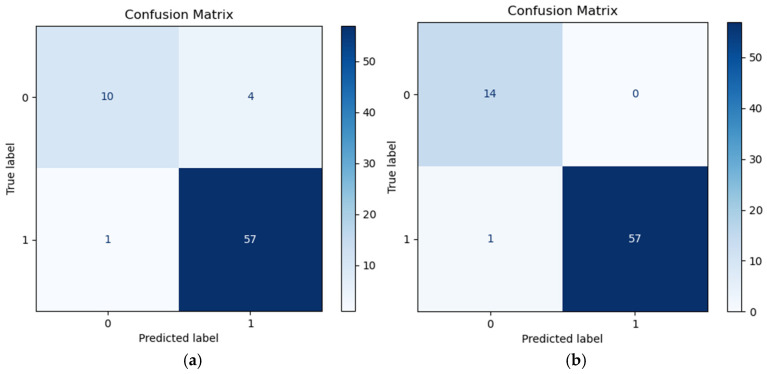
Confusion matrix (**a**) Time and frequency domain + SVM; (**b**) Proposed.

**Figure 14:**
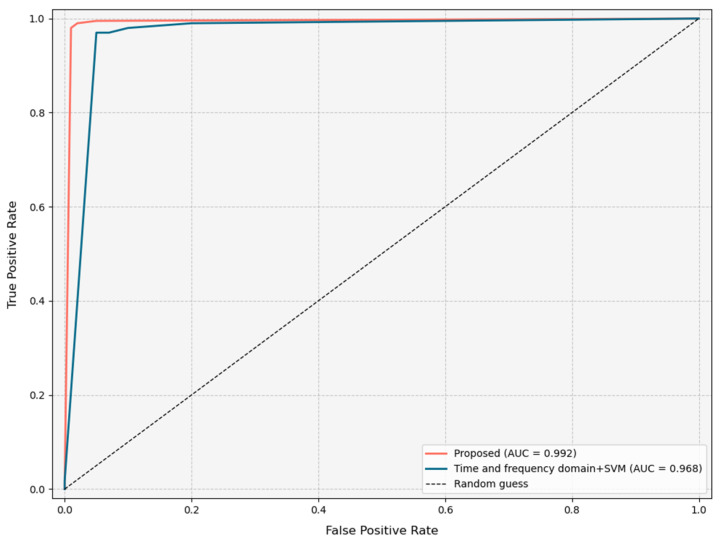
ROC curve.

As shown in Figure 13 and Table 4, during the model training process, we conducted a comprehensive evaluation of the model’s performance using multiple metrics such as confusion matrix, accuracy, recall, and F1 score. The experimental results indicate that the SVM model based on the features proposed in this paper performs excellently in the task of classifying pipeline leakage signals, achieving an accuracy of 98.6%, significantly higher than the 93% accuracy of time-frequency domain features reported in the literature. Furthermore, our method also outperforms the comparison methods in key metrics such as recall and F1 score. Figure 14 presents a comparison of the ROC curves for the two types of features, showing that the ROC curve for the features proposed in this paper is closer to the top left corner, indicating a significantly better classification performance than the time-frequency domain features in the literature. In summary, the SVM model using the features proposed in this paper as input vectors demonstrates high accuracy and reliability in the classification task of leakage and non-leakage signals, effectively identifying pipeline leakage phenomena.

### 5.2. Field Test Results

Based on the analysis above, the feature extraction method proposed in this paper can effectively identify leaks. To this end, the authors conducted on-site leak tests in the water supply pipeline and compared the time-frequency domain features extracted in [30]. In this comparison, 40 groups of leak cases and 40 groups of non-leak cases were used as test subjects.

From Table 5, it can be seen that during on-site testing, for the same pipeline under conditions of leakage or non-leakage, the leak detection rate of the time-frequency domain features proposed in the literature is 10%, and the false detection rate is 7.5%, both of which are higher than the 5% and 0 of the feature extraction method proposed in this paper. Therefore, the frequency domain features proposed in this paper can be better applied to pipeline leakage identification.

## 6. Conclusions

This article focuses on the identification of micro-leakages in pipelines and establishes a method for calculating a mixed model of flow field and acoustic field. It summarizes the characteristics of the flow field and acoustic field under different operating conditions and extracts signal features with significant differences for leak detection. Compared with previous CFD–CAA-based studies, the proposed LES–CAA–SVM hybrid framework achieves a complete linkage from leakage mechanism analysis to identification modeling. The high consistency between simulation results and experimental measurements within the main frequency band demonstrates the higher physical fidelity of this method relative to traditional simplified models. Meanwhile, the SVM classifier shows superior accuracy and generalization performance under small-sample conditions compared with conventional statistical models and some neural-network-based approaches. The following conclusions can be drawn:

1. The acoustic signals from pipeline leaks are influenced by different pressure fluctuations and velocity impacts caused by various operating conditions. An increase in pipeline pressure exacerbates changes in the flow field at the leak site, and the aperture will also increase the jet flow and turbulence intensity. Additionally, the diameter and material of the pipeline directly affect the frequency range that the sensors can capture.
2. The acoustic signals from pipeline leaks primarily appear as wideband noise in the range of 500–3000 Hz, with the main peak for carbon steel concentrated around 1000 Hz, which may fluctuate based on different operating conditions. Increases in pressure and the size of the leak hole will lead to a slight increase in the main frequency.
3. The actual signal patterns measured in experiments validate the guidance provided by simulations. The patterns and parameters obtained from simulations provide a theoretical basis for feature extraction. The leak detection model constructed based on the Support Vector Machine (SVM) algorithm demonstrates high accuracy and reliability, achieving a precision of 98.6%, effectively identifying leak phenomena in pipelines. Nevertheless, certain limitations remain. The current simulations rely on simplified pipeline geometries and a limited set of operating conditions, and the fluid–structure-acoustic coupling adopts a one-way transfer mechanism without accounting for reverse feedback. In addition, the experimental dataset is relatively small, and model robustness under complex real-world conditions requires further verification. Future work will expand the simulation parameter space, incorporate more complex boundary and flow conditions, integrate deep-learning-based adaptive feature extraction, and develop a real-time online leak monitoring prototype system based on the proposed framework.

## Figures and Tables

**Table 1:** Parameter table of operational conditions.

Parameter	Range
Pipe pressure, MPa	0.2–4
Pipe inner diameter, mm	10–800
Pipe length, mm	100–2400
Leakage diameter, mm	0.1–2
Pipe wall thickness, mm	DN < 100:1
	DN > 100:4
Internal pipe medium	Water
Medium temperature, °C	25
Fluid velocity, m/s	4
External pipe medium	Air
Pipe material	B235 carbon steel, 304Stainless steel, PU

**Table 2:** Grid independence verification.

Mesh	Number of Grid Cells	Mass Flow
Mesh1	1,969,117	30.2
Mesh2	2,207,471	37.5
Mesh3	3,026,813	36.8
Mesh4	3,317,651	36.7
Mesh5	3,670,383	36.8

**Table 3:** Ablation study of feature combinations on SVM classification accuracy.

Feature Combination	Accuracy (%)
All three features	98.612
Centroid frequency + Frequency entropy	80.339
Energy ratio + Frequency entropy	91.264
Energy ratio + Centroid frequency	94.211
Energy ratio only	90.512

**Table 4:** Performance of different models’ parameters.

ML Model	Accuracy	Recall
Proposed	98.611	98.276
Time and frequency domain +SVM	93.056	97.276

**Table 5:** Identification results of leakages and non-leakages.

	Time and Frequency Domain +SVM		Proposed	
	non-Leakages	leakages	non-Leakages	leakages
Number of tests	40	40	40	40
Correct	37	36	38	40
Missing rate		10.0%		0
False drop rate	7.5%		5.0%	
Accuracy	91.25%		97.5%	

## Data Availability

The data presented in this study are available on request from the corresponding author.
